# A Systems Biology Approach Reveals Converging Molecular Mechanisms that Link Different POPs to Common Metabolic Diseases

**DOI:** 10.1289/ehp.1510308

**Published:** 2015-12-18

**Authors:** Patricia Ruiz, Ally Perlina, Moiz Mumtaz, Bruce A. Fowler

**Affiliations:** 1 Computational Toxicology and Methods Development Laboratory, Division of Toxicology and Human Health Sciences, Agency for Toxic Substances and Disease Registry, Atlanta, Georgia, USA; 2 Sanford Burnham Prebys Medical Discovery Institute, La Jolla, California, USA; 3 Emory University Rollins School of Public Health, Atlanta, Georgia, USA

## Abstract

**Background:**

A number of epidemiological studies have identified statistical associations between persistent organic pollutants (POPs) and metabolic diseases, but testable hypotheses regarding underlying molecular mechanisms to explain these linkages have not been published.

**Objectives:**

We assessed the underlying mechanisms of POPs that have been associated with metabolic diseases; three well-known POPs [2,3,7,8-tetrachlorodibenzodioxin (TCDD), 2,2´,4,4´,5,5´-hexachlorobiphenyl (PCB 153), and 4,4´-dichlorodiphenyldichloroethylene (p,p´-DDE)] were studied. We used advanced database search tools to delineate testable hypotheses and to guide laboratory-based research studies into underlying mechanisms by which this POP mixture could produce or exacerbate metabolic diseases.

**Methods:**

For our searches, we used proprietary systems biology software (MetaCore™/MetaDrug™) to conduct advanced search queries for the underlying interactions database, followed by directional network construction to identify common mechanisms for these POPs within two or fewer interaction steps downstream of their primary targets. These common downstream pathways belong to various cytokine and chemokine families with experimentally well-documented causal associations with type 2 diabetes.

**Conclusions:**

Our systems biology approach allowed identification of converging pathways leading to activation of common downstream targets. To our knowledge, this is the first study to propose an integrated global set of step-by-step molecular mechanisms for a combination of three common POPs using a systems biology approach, which may link POP exposure to diseases. Experimental evaluation of the proposed pathways may lead to development of predictive biomarkers of the effects of POPs, which could translate into disease prevention and effective clinical treatment strategies.

**Citation:**

Ruiz P, Perlina A, Mumtaz M, Fowler BA. 2016. A systems biology approach reveals converging molecular mechanisms that link different POPs to common metabolic diseases. Environ Health Perspect 124:1034–1041; http://dx.doi.org/10.1289/ehp.1510308

## Introduction

Persistent organic pollutants (POPs) are ubiquitous environmental contaminants. They include polychlorinated dibenzo-*p*-dioxins (PCDDs), polychlorinated dibenzofurans (PCDFs), polychlorinated biphenyls (PCBs), and organochlorine pesticides. 2,3,7,8-Tetrachlorodibenzodioxin (TCDD), a representative of the dioxin chemical family, is unintentionally produced during chlorine bleaching processes, drinking water chlorination, and incineration processes [Agency for Toxic Substances and Disease Registry (ATSDR) 2012]. 4,4´-Dichlorodiphenyldichloroethylene (*p,p*´-DDE) is a metabolite of DDT that has been used as an insecticide for insect vectors of malaria and typhus (ATSDR 2008). Polychlorinated biphenyls (PCBs) are industrial chemicals principally used as heat exchange fluids in transformers and capacitors that were banned in the United States in 1977 (ATSDR 2011).

Epidemiological studies have reported associations between POPs and metabolic diseases such as Type 2 diabetes (T2D), obesity, and metabolic syndrome, but the potential underlying mechanism(s) are not known (Langer et al. 2014; Lee et al. 2006, 2010, 2011; Rylander et al. 2005; Suzuki et al. 2008). The three POPs evaluated in the present study (TCDD, PCB 153, and *p,p*´-DDE) have been associated with metabolic disorders in observational studies, but the potential molecular mechanisms that might underlie endocrine disruption and disease development are far from understood (Everett et al. 2007; Henriksen et al. 1997; Lee et al. 2014; Longnecker and Michalek 2000; Magliano et al. 2014; Rignell-Hydbom et al. 2007; Turyk et al. 2009; Wang et al. 2008).

Because metabolic diseases are increasing in frequency throughout the world, further investigation and understanding of the possibility that exposure to POPs contributes to the etiology of diabetes, obesity, and cardiovascular disease is critical (Taylor et al. 2013; Thayer et al. 2012). Metabolic syndrome may affect up to 1 in 5 people, and its prevalence increases with age (Paoletti et al. 2006). It is estimated that ≤ 25% of the U.S. population has metabolic syndrome (Ford et al. 2004).

Researchers have hypothesized that low-level POP exposure can cause metabolic changes through a network of pathways, including increased insulin resistance and obesity preceding the development of T2D (Barouki et al. 2012; Barrett 2013; Lee et al. 2014; Taylor et al. 2013). Within this network, different POPs might also cause metabolic syndrome through slightly overlapping pathways to cause disturbances in glucose homeostasis. Such disturbances include inhibition of insulin action and induced down-regulation of master regulators of lipid homeostasis. The situation is further complicated by the realization that POP-induced alterations in epigenetic regulatory mechanisms may occur during sensitive developmental periods and lead to diseases such as obesity and T2D later in life (Barouki et al. 2012).

In toxicology, systems biology facilitates the identification of important pathways and molecules from large data sets. These tasks can be extremely laborious when performed using a classical literature search. Computational systems biology offers more advantages than simply providing a high-throughput literature search engine; these tools may provide the basis for establishing hypotheses on potential links between environmental chemicals and human diseases. Comprehensive databases containing information on networks of human protein–protein interactions and protein–disease associations make it possible to identify such links. Experimentally determined target data for the specific chemical of interest can be uploaded and superimposed on these networks to obtain additional information that can be used to establish hypotheses on links between the chemical and human diseases. Such information can also be used to design rational animal- and cell-based laboratory experiments to test the established hypotheses.

In this study, we examined potential linkages for combined exposures to three specific POPs, cellular pathway alterations, and metabolic disturbances related to the development of important clinical outcomes. We used an integrated global approach that brought together *a*) predictive chemical analyses based on compound structure and *b*) knowledge bases of chemogenomics data associating compounds with biological and toxicological properties. We then performed an *in silico* evaluation of the possible joint effects of POPs on metabolic pathways that could lead to metabolic diseases. We sought to discover common downstream activation targets for all three POPs as a mixture. Although inhibitory targets were also analyzed, we chose to focus on the genes that could ultimately be up-regulated and lead to increased abundance on the protein level. The rationale for this focus was to set the stage for the discovery of screening biomarkers, particularly those present in easily accessible tissues/fluids, the accessibility of which could be improved when increased in abundance, as opposed to depleted. It is hoped that these data will stimulate the formation of new, testable hypotheses to address some of the data gaps previously identified by Barrett (2013), La Merrill et al. (2013), and Taylor et al. (2013).

## Methods

Three POPs (*p*,*p*´-DDE, TCDD, and PCB 153) were selected for investigation in this study because they are commonly detected in the environment and in human tissues. Based upon data from the epidemiological and data mining literature noted above, they have also been linked with metabolic diseases such as T2D (Everett et al. 2007; Henriksen et al. 1997; Lee et al. 2010; Longnecker and Michalek 2000; Turyk et al. 2009; Wang et al. 2008).

The majority of available POP studies have focused on these three POPs on an individual basis. To our knowledge, there are no published studies on their combined potential interactive effects at the molecular level in relation to clinical disease outcomes.

### The Pathway Analysis Tools: Metacore™/Metadrug™

**Figure 1:**
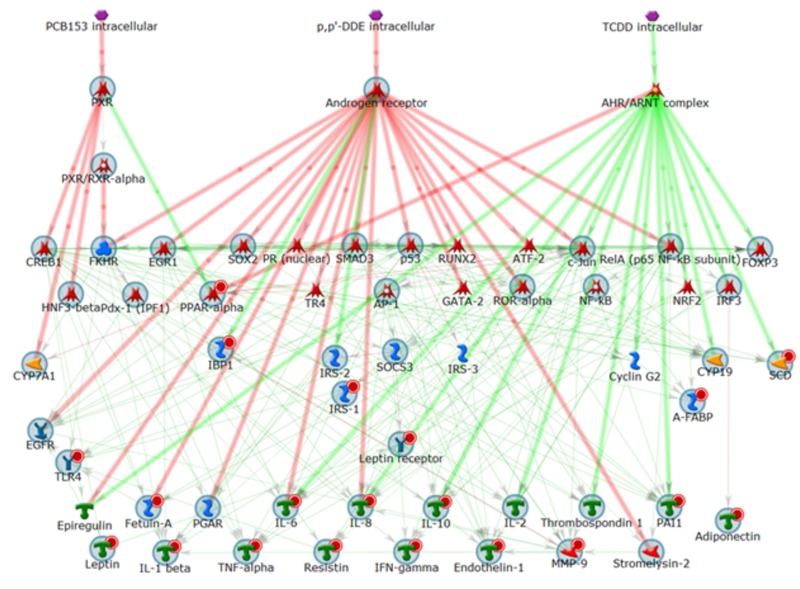
Proposed global network for potential converging genes associated with diabetes/insulin resistance, obesity, or metabolic syndrome X, and the three POPs. Thick lines highlight the closest interactions. Large gray circles represent union genes. Small red circles indicate the intersection genes for the three diseases. Symbols defined by MetaCore™ at http://lsresearch.thomsonreuters.com/static/uploads/files/2014-05/MetaCoreQuickReferenceGuide.pdf: green arrows, activating interactions; red arrows, inhibiting interactions; POPs, purple hexagons; catalytic factors, yellow; transcription factors, red; cytokines and lipoproteins, green; receptors and adaptor proteins, blue.

The molecular structure (.mol) files of three POPs [*p*,*p*´-DDE, TCDD, and PCB 153 (Figure 1)] were separately uploaded to MetaCore™/MetaDrug™, a proprietary systems biology software solution (Thomson Reuters; originally developed by GeneGo, Inc.). This software is built on a proprietary database (MetaBase™) to allow functional and network analysis of primary and secondary effects of any query compound in the context of manually curated molecular interactions and pathways (Ekins et al. 2007).

MetaCore™/MetaDrug™ are analytical tools built on top of a manually curated database of literature findings that support various types of molecular interactions and ontologies, including disease relationships. These tools help a user to analyze information from experimental results or to mine the underlying content from the MetaBase™ database content directly.

Advanced Search is a java application tool in MetaCore™ that facilitates searching combined information, for example, “find all compounds that inhibit EGFR with IC_50_ < 1 μM.” Using Advanced Search allows us to create a Boolean query and to retrieve the results. A detailed methodological description of the systems biology procedures and protocols for using the software are available (http://lsresearch.thomsonreuters.com/).

When querying content from MetaBase™, Advanced Search allows us to differentiate low- and high-trust annotation information behind interactions. “High-trust” content has been confirmed to have published small-scale experimental evidence (e.g., co-immunoprecipitation plus luciferase reporter assay). Low-trust interactions have only been validated via high-throughput screening/co-expression or predictive analysis studies and lack more rigorous experimental evidence. Molecular entities can affect a target directly and indirectly. Mechanisms used to describe direct physical interactions include binding, covalent modification, phosphorylation, and so on. Indirect mechanisms include influence on expression, co-regulation of transcription, unspecified, and others, as stated in the legend. Only high-trust direct interactions with known effects (activation or inhibition) were used in this study.

### POP Pathway Analysis

To map all of the possible pathways from the three selected POPs to their downstream targets, primary targets (i.e., the targets of direct chemical actions that lead to a response in the cells of the mammalian organism; all other targets of the chemical are considered secondary) were first determined using MetaCore™ and MetaDrug™ content. Of those primary targets, only direct binding targets that had further downstream interactions were considered. To reduce the complexity presented by primary targets that already participate in thousands of annotated molecular interactions, the Advanced Search tool was used. This tool allowed the construction of direct database queries of interactions leading from primary/direct targets of each POP to common targets shared by all three POPs in three or fewer steps and with an inferred activating effect. These conditions meant that from the compound itself, the allowed network depth/distance would be three or fewer steps. The focus on downstream activation targets was presumed to be of greatest utility if such targets were to be used for detection as biomarkers. All combinations of path lengths within three interactions were considered. For example, some targets may have been two steps downstream of one POP but three steps downstream of the other two POPs. For all of the multi-step paths, the assumption was made that to achieve downstream activation, not only activation interactions but also inhibitory interactions could be considered, provided that they added up to the final effect of activation. For example, inhibition of an inhibitor can result in subsequent activation. Thus, all combinations of the following interaction paths were considered in our search queries:

Two-step paths:
  activation, activation
  inhibition, inhibition
Three-step paths:
  activation, activation, activation
  activation, inhibition, inhibition
  inhibition, inhibition, activation
  inhibition, activation, inhibition

Note that there were no common primary targets for all three POPs; hence, no one-step paths were obtained in this analysis.

The resulting combined list of potential common activation targets was used for network construction. The “shortest paths” network-building option was used. The maximum number of interaction steps is defined by the user from a range of 1 to 10 interaction steps and was set to three as a maximum in this study; the algorithm attempts to build the shortest direct paths between selected objects using up to the maximum number of interactions defined by the user. This operation yielded the resulting interconnected network diagram. Interaction effects were then checked for concordance (or agreement, to make sure there were no conflicting interactions). Only direct activation or inhibition interactions were used for network construction. “Direct” refers to small-scale molecular physical interactions, as described earlier, the effects of which are either activation or inhibition, meaning that interactions that are indirect or that have an unspecified effect would not be considered by the shortest-paths algorithm. Only concordantly regulated molecules (also referred to as network nodes) were displayed on the final network, with only those interactions that led to downstream activation. Therefore, the terminal nodes (those genes/proteins that only had upstream interactions) had only those sequences of interactions leading toward them that would result in activation. Even though advanced queries produced target lists (queries retrieve lists of genes), network building was still needed for a visual representation of the interaction space that met the requirements of the queries and mechanistically tied the POPs to the targets. Network building was also needed to manually check and remove any signaling conflicts that arose between intermediate nodes along the queried paths. For example, if gene A is a logical linker downstream of a POP target and its downstream common activation target but is regulated in the opposite direction by the primary target of another POP, then a mixed message would result, and gene A would have to be removed from the final network. In other words, a mixed message occurs when an intermediate protein receives a signal from a POP through its target to behave in one way (for example, to activate or induce signaling), but through a second target for another POP, the effect is in opposition (to inhibit or suppress signaling); thus, the intermediate protein receives conflicting signals.

The final network was narrowed to only those downstream targets associated with the following: diabetes/insulin resistance (IR), obesity, and metabolic syndrome X. Finally, to clarify the specific signaling paths, the final network was subdivided into subnetworks with smaller portions of information based on one downstream target at a time for increased resolution.

## Results

### Interaction Queries and Network Construction

Starting with the three nodes that represented TCDD, PCB 153, and *p,p*´-DDE, we identified primary targets using advanced database search queries, followed by directional network construction. Because these three POPs are so structurally different, they bind different primary targets: the pregnane X receptor (PXR) for PCB 153, the androgen receptor (AR) for *p,p*´-DDE, and the aryl hydrocarbon receptor (AhR) for TCDD. The difference in targets suggested different modes of action and downstream effects for these three chemicals. However, with the addition of only one or two more interaction steps, the literature-based pathway reconstruction/modeling approach made it possible to determine which activation targets of one or two of these compounds could also be activated by the third compound. Such converging common activation targets were identified from cumulative results obtained from the Advanced Search queries: 349 concordance targets were identified in three steps or fewer (data not shown) and had known positive disease association. Only high-trust direct physical interactions of known effects (activation or inhibition) were used for the queries and for network building. Networks were built downstream of all three POPs using three steps or fewer for Dijkstra’s shortest paths algorithm (Dijkstra 1959). All interaction effects were then checked for concordance on the final network representation.

### Proposed Final Network

The connectivity of the network revealed that six targets [interleukin 6 (IL-6), IL-8, RelA (p65), c-Jun, FKHR, and Cyclin D1] were activated by the three POPs in 2.33 steps on average (two steps from two of the POPs and one additional step from the third POP (thus, three steps from the third POP); therefore, the average of each set of chemical steps was (2 + 2 + 3)/3 = 2.33. The network also showed that 35 targets were activated by the three POPs in 2.67 steps on average [two steps from one of the chemicals and one additional step from the two others, (2 + 3 + 3)/3 = 2.67], yielding a total of 41 targets that could be activated by the three POPs in fewer than three network interaction steps on average.

The complete final network (Figure 1) reveals the genes associated with diabetes/insulin resistance, or obesity, or metabolic syndrome X (union, large gray circles around gene icons). The genes that also have a small circle (tagging the gene’s icon on the top right) represent the annotated associations with all three diseases (intersection). Based on this systems biology–generated global network (Figure 1), a joint mechanism of action was proposed for the combined exposure to the three POPs and for the additive effects that may be anticipated. Figure 1 shows some intermediate cross-interactions between the downstream targets (shown in green). These common activation processes can influence or activate each other and imply even more functional and mechanistic connectivity/synergy. Although this systems biology–generated global network cannot be considered as proof of causal linkages without further experimental validation, it provides justification for the mechanistic hypothesis and contributes new information potentially linking available published toxicology and disease information domains.

### Delineating Subpathways from the Network

**Figure 2:**
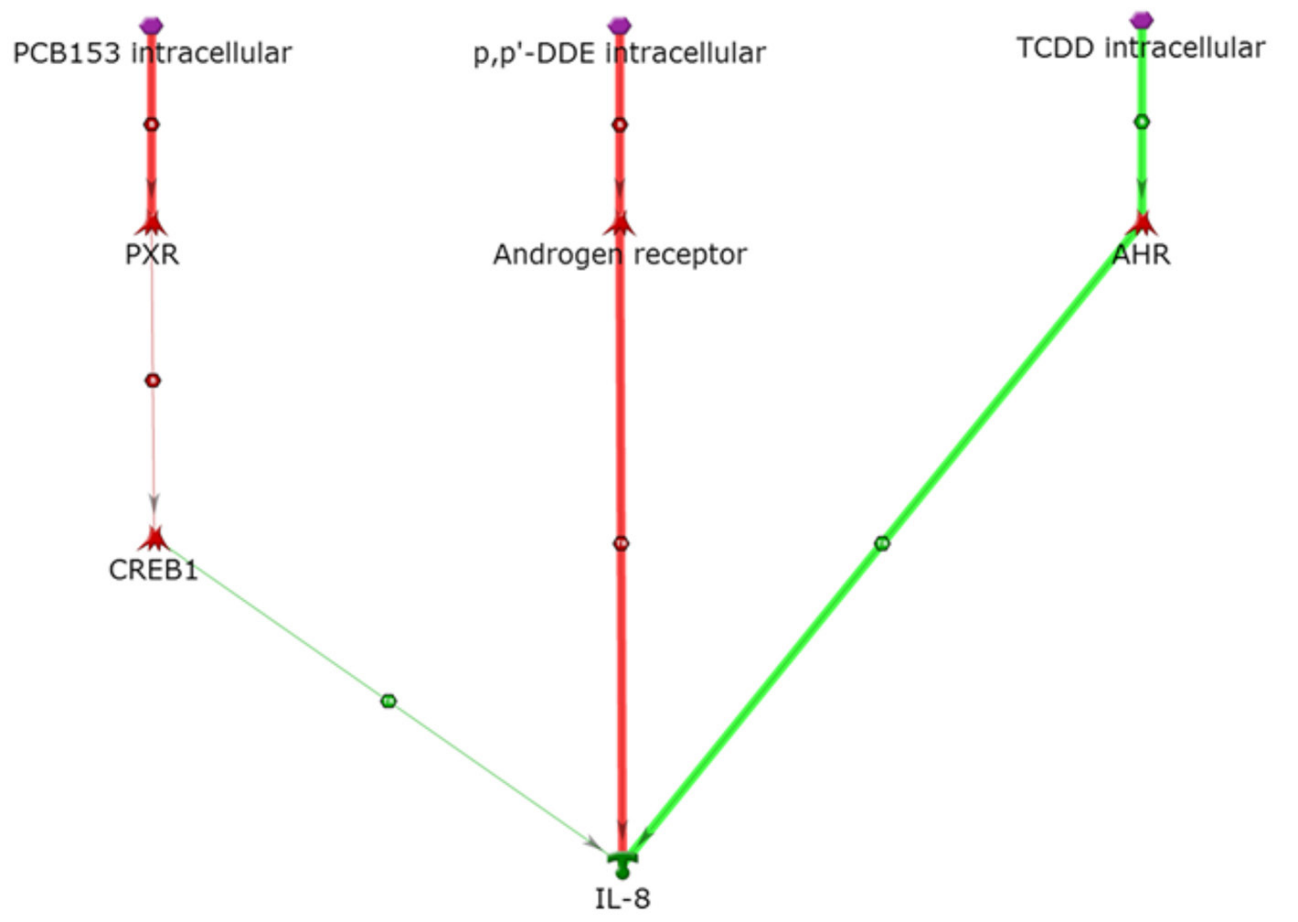
Activation of IL-8. Thick lines highlight the closest interactions, and thin lines indicate intermediate and farther interactions. AHR, aryl hydrocarbon receptor; PXR, pregnane X receptor. Symbols as defined by MetaCore™ at http://lsresearch.thomsonreuters.com/static/uploads/files/2014-05/MetaCoreQuickReferenceGuide.pdf: green arrows, activating interactions; red arrows, inhibiting interactions; POPs, purple hexagons; IL-8, green symbol; transcription factors, red symbols.

To clarify specific signaling pathways, the final global network was divided into subnetworks with smaller portions of information for increased resolution. For example, IL-8 can be activated by *p,p*´-DDE via the AR (inhibition of an inhibitor) and activated by TCDD via the AhR (activation of an activator) (Vogel et al. 2007) (Figure 2). PCB 153 is not known to activate IL-8 directly or through its primary target (the PXR). However, with evaluation of one additional step, a plausible mechanism was revealed: CREB1 can be activated by PCB 153 through the PXR (inhibition of an inhibitor) via documented direct binding interactions (Kodama et al. 2007; Tabb et al. 2004). CREB1 can then lead to activation of IL-8 via well-documented promoter binding (Mayer et al. 2013) (Figure 2). The potential link between IL-8 and PCB 153 is noteworthy because it establishes a link between PCB 153 and IL-8 through CREB1 by allowing one immediate step.

**Figure 3:**
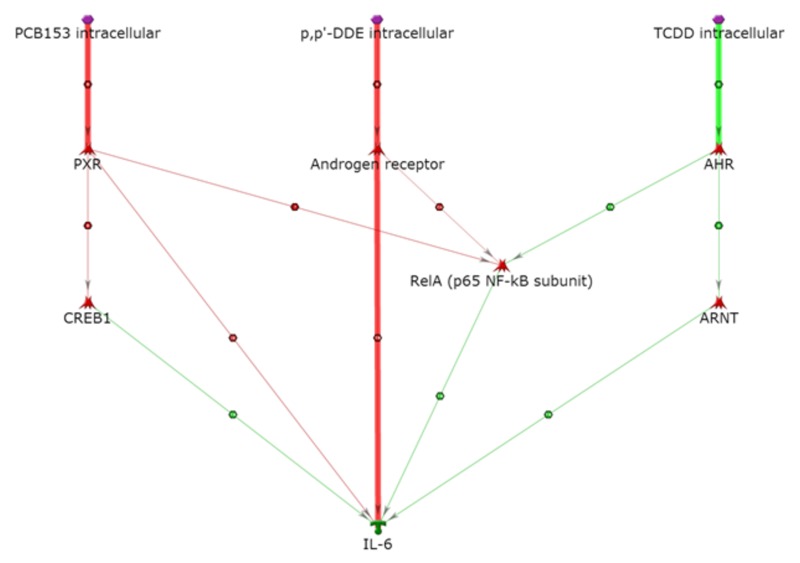
Activation of IL-6. Thick lines highlight the closest interactions, and thin lines indicate intermediate and farther interactions. AHR, aryl hydrocarbon receptor; PXR, pregnane X receptor. Symbols defined by MetaCore™ at http://lsresearch.thomsonreuters.com/static/uploads/files/2014-05/MetaCoreQuickReferenceGuide.pdf: green arrows, activating interactions; red arrows, inhibiting interactions; POPs, purple hexagons; IL-6, green symbol; transcription factors, red symbols.

IL-6 is well known to promote inflammation and proinflammatory effects (Scheller et al. 2011). As illustrated in Figure 3, our analysis suggests that that IL-6 can be directly activated by TCDD via the AhR/ARNT complex (activation of an activator); AhR and ARNT are close neighbors of IL-6 that often act together in many pathways. AhR can also activate RelA, which can activate IL-6. PCB 153 and *p,p*´-DDE can inhibit RelA (inhibition of an inhibitor) via PXR and AR, respectively, and then RelA activates IL-6. There is a direct pathway for activation of IL-6 from *p,p*´-DDE via the AR (inhibition of an inhibitor). IL-6 could be activated from PCB 153 in the same pathway as that described for IL-8 (Kodama et al. 2007; Tabb et al. 2004).

**Figure 4:**
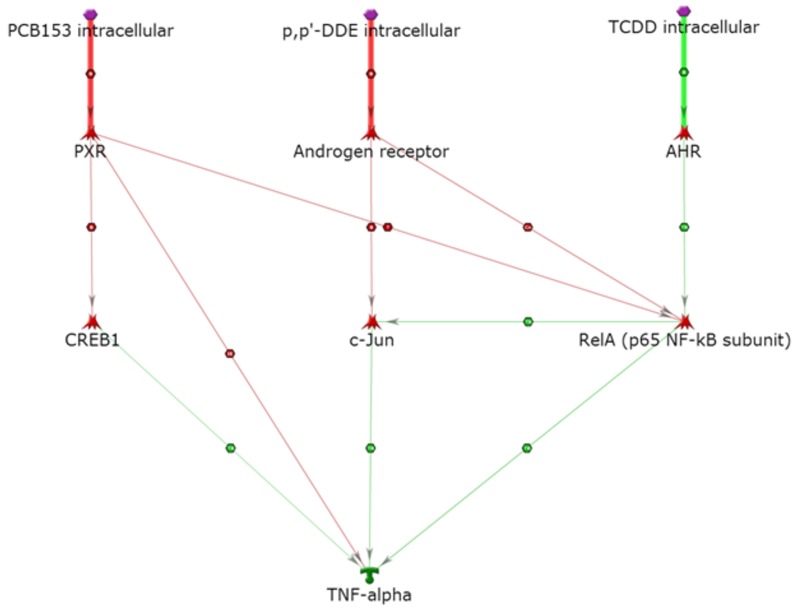
Activation of TNF-α. Thick lines highlight the closest interactions, and thin lines indicate intermediate and farther interactions. AHR, aryl hydrocarbon receptor; PXR, pregnane X receptor. Symbols defined by MetaCore™ at http://lsresearch.thomsonreuters.com/static/uploads/files/2014-05/MetaCoreQuickReferenceGuide.pdf: green arrows, activating interactions; red arrows, inhibiting interactions; POPs, purple hexagons; TNF-α, green symbol; transcription factors, red symbols.

As shown in Figure 4, our analysis suggests that tumor necrosis factor-alpha (TNF-α) can be activated by TCDD via AhR and RelA (activation of an activator), where RelA directly activates TNF-α; *p,p*´-DDE can also activate TNF-α via the AR and c-Jun (inhibition of an inhibitor). PCB 153 activates TNF-α through the same pathways as those described for activation of IL-6 and IL-8. PXR (inhibition of an inhibitor) also provides a direct path for activation of TNF-α from PCB 153. CREB1 could be an important link to these cytokine activation pathways. RelA could represent a common step in the activation of TNF-α by all of these individual POPs. c-Jun plays an important role in all of the inflammation pathways, and together with RelA, promotes the inflammation pathway (Ip and Davis 1998; Tak and Firestein 2001).

**Figure 5:**
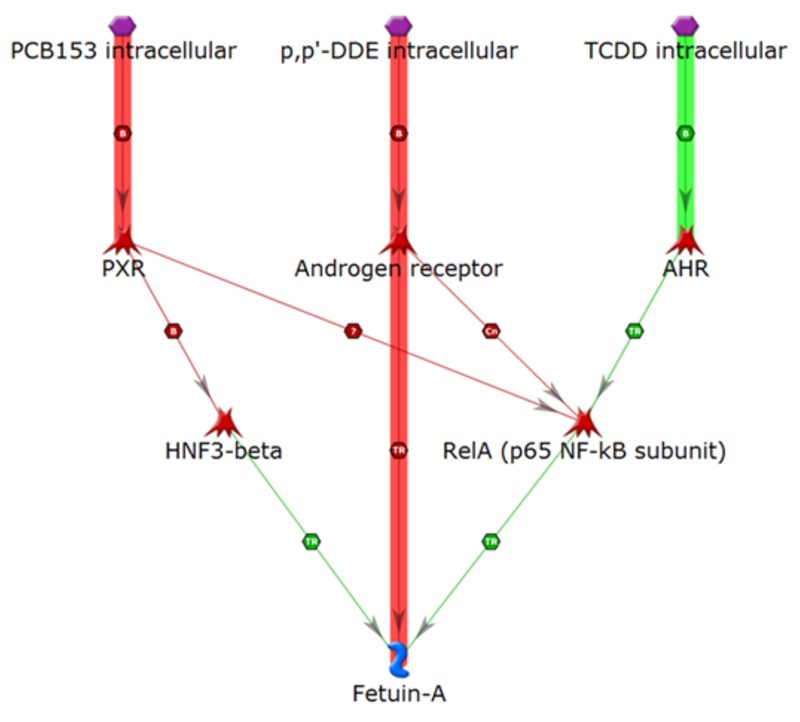
Activation of fetuin A. Thick lines highlight the closest interactions, and thin lines indicate intermediate and farther interactions. AHR, aryl hydrocarbon receptor; PXR, pregnane X receptor. Symbols defined by MetaCore™ at http://lsresearch.thomsonreuters.com/static/uploads/files/2014-05/MetaCoreQuickReferenceGuide.pdf: green arrows, activating interactions; red arrows, inhibiting interactions; POPs, purple hexagons; Fetuin A, blue symbol; transcription factors, red symbols.

As illustrated in Figure 5, our analysis suggests that fetuin A can be activated by TCDD via the AhR and RelA (activation of an activator), and then RelA directly activates fetuin A. PCB 153 and *p,p*´-DDE can inhibit RelA (inhibition of an inhibitor) via the PXR and the AR, respectively, and then RelA can activate fetuin A. Again, it is a noteworthy observation that RelA could represent a common step in the activation of fetuin A, IL-6, and TNF-α by all of these POPs. Thus, the counterpart proinflammatory effects of these proteins that are activated through intracellular signaling pathways may involve RelA.

**Figure 6:**
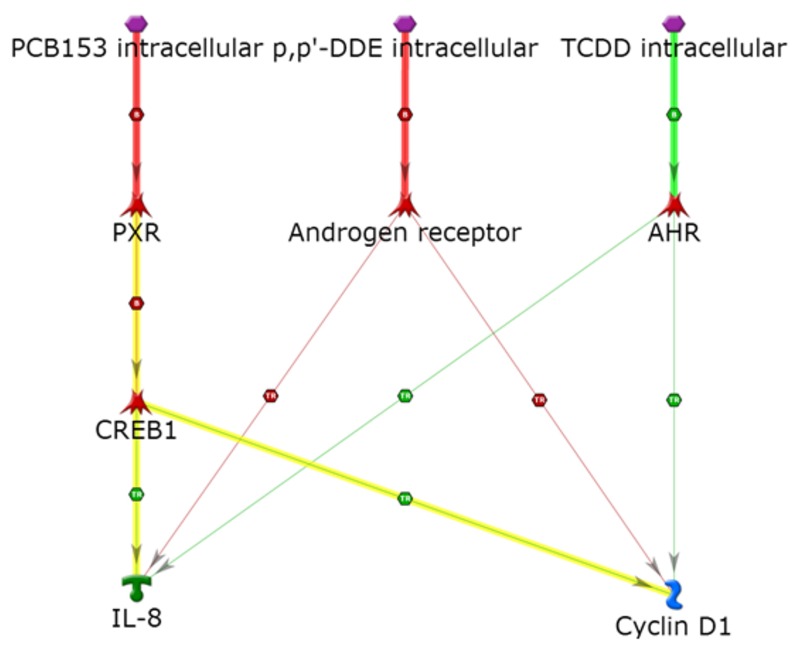
Activation of IL-8 and cyclin D1: proposed POP mixture pathway shared by inflammation and cancer. Thick red and green arrows emphasize primary POP binding targets with inhibiting and activating effects, respectively. Yellow highlight indicates paths that needed an additional node (CREB1) to further converge on the same downstream targets via the PXR, whereas the AR and the AHR directly connected to the common downstream targets. AHR, aryl hydrocarbon receptor; PXR, pregnane X receptor. Symbols defined by MetaCore™ by Thomson Reuters http://lsresearch.thomsonreuters.com/static/uploads/files/2014-05/MetaCoreQuickReferenceGuide.pdf: green arrows, activating interactions; red arrows, inhibiting interactions; POPs (TCDD, PCB 153 and *p*,*p*´-DDE), purple hexagons, transcription factors, red symbols; cyclin D1, blue symbol; IL-8, green symbol.

Overall, the whole resulting network is populated with a combination of metabolic genes, insulin signaling, immune response signaling, and the inflammation cascade of cytokines and transcription factors. Based on our analysis, we hypothesize that common pathways that converge through the cytokines may contribute to inflammatory processes that may lead to metabolic diseases via circulation and via creation of a chronic inflammatory background in adipocytes and in liver and pancreatic tissues. These circumstances can lead to adipogenesis, pancreatic β-cell dysfunction, insulin resistance, glucose intolerance, liver disease, and inability to cope with increased dietary intake, which over time can result in the development of serious metabolic disease phenotypes. In addition to inflammation, some cancer-associated targets are also present. For example, as shown in Figure 6, our analysis suggests that cyclin D1 and IL-8 share common pathways. PCB 153 requires three steps to reach cyclin D1 (through PXR and CREB1); it requires the same intermediate steps to reach IL-8. Furthermore, cyclin D1 is activated by TCDD though the AhR, as is IL-8. Similar steps occur with the direct transcription methylation of the AR, which is then inhibited though *p,p*´-DDE. Thus, based on our analysis, we hypothesize that there is an overlap of mechanisms between inflammatory processes and cancer development and progression that increases the potential for the carcinogenicity of mixtures of these POPs.

## Discussion

By integrating the available information and bridging the gap between toxicology, epidemiology, and chemistry within the world of disease mechanisms, we can look beyond the primary targets of the individual POPs to two or three steps down the relevant pathway. Analysis of molecular networks and all possible downstream targets is extremely complex. Our approach, based on mechanistic annotated networks, allows identification of common targets that are beyond the primary targets. Although molecular interaction data have been reported for individual POPs and have been confirmed by published experimental studies (Goldberg 2009, Kuwatsuka et al. 2014), to our knowledge, the data have not been previously integrated for a mixture of these compounds in the step-by-step continuum and sequential manner described here.

For example, *p,p*´-DDE, TCDD, and PCB 153 can act as agonists or antagonists of the AR, the AhR, and the PXR, respectively (ATSDR 2008, 2011, 2012). Thus, these chemicals are of specific concern for developing organisms that are highly sensitive to hormonal changes, and exposure to these chemicals is critical because it could lead to permanent changes throughout life. These POPs might act over time at low levels of exposure during fetal or early-life periods and have a particular impact on health. The finding of potential human health effects from interactions of multiple chemicals creates many difficulties, and there is a great need for reliable biomarkers of effects and of exposure. Nevertheless, recent reports support the notion that documented interactions downstream of the POPs implicate each POP in perturbation of pathways that might lead to various metabolic diseases such as obesity and T2D (Scrivo et al. 2011).

A close look at the nuclear receptor signaling pathway revealed that PCB 153, TCDD, and *p,p*´-DDE have overlapping and interconnected pathways that have the potential to cause a variety of biological perturbations. The AhR directly activates and transcriptionally regulates expression of IL-8 (Vogel et al. 2007), and IL-8 and TCDD were associated with diabetes in a cross-sectional analysis of data from an NHANES cohort (Lee et al. 2006). Our systems biology analysis suggests a link between PCB 153 and IL-8 through CREB1 by one intermediate step from its primary target. The potential link between IL-8 and PCB 153 is noteworthy. CREB1 could be an important link to cytokine activation pathways. Based on our analysis, we can hypothesize a joint toxic action pathway (involving IL-8 as well as other cytokines) for mixtures of these three specific POPs that could be experimentally tested and extended to other POPs.

Various toxic compounds may trigger abnormal inflammatory responses directly or indirectly by interfering with the normal physiological functioning of cells or tissues (Medzhitov 2008). These effects could play a role in the development of insulin resistance and diabetes. Kim et al. (2014) analyzed the influence of POP concentrations on insulin resistance in a cross-sectional study of nondiabetic individuals, most of whom had cancer. In a cross-sectional study of 39 caucasians and 72 First Nations adults, Imbeault et al. (2012) reported a weak but significant association of elevated levels of POPs with cytokines. Studies on POPs and human adipose cells showed that precursor cells and adipocytes were targets of POPs and that these pollutants mainly triggered the inflammation pathway (Kim et al. 2012). In a Japanese study involving 40 patients from the Yusho poisoning incident and 40 controls, Kuwatsuka et al. (2014) found that serum levels of certain interleukins (IL-17, IL-1β, and IL-23) and of TNF-α were higher in patients who had been exposed to POPs, including PCBs, through consumption of contaminated rice oil. Circulating inflammatory biomarkers such as C-reactive protein (CRP), IL-6, TNF-α, monocyte chemotactic protein 1 (MCP 1), intercellular adhesion molecule 1 (ICAM 1), vascular cell adhesion protein 1 (VCAM 1), and E-selectin have been associated with a variety of metabolic disorders and their associated outcomes (Goldberg 2009). However, in a study population of 72 participants, Pal et al. (2013) reported no significant association between POP concentrations and markers of insulin resistance when comparing diabetic and nondiabetic individuals in a Northern Ontario, Canada population. Similarly, in a cross-sectional study of 1,016 individuals (all 70 years of age) from Sweden, Kumar et al. (2014) observed no association between levels of POPs and proinflammatory cytokines (IL-6, MCP-1, and TNF-α). Differences in the results between studies could be attributable to various factors, including the number of individuals in the studies, the presence of other diseases, gut microbiota, diet composition, early-life nutrition, and noncausal associations as a result of confounding or other sources of bias.

As noted previously, numerous studies have shown connections between cytokines and metabolic disease, cytokine levels and POPs, and POP levels and metabolic diseases. However, few give a clear articulation of the underlying mechanisms, particularly for mixtures of similar and dissimilar chemicals. In the study of disease biology and the pathogenesis of disease, much effort is given to elucidating and validating new pathways. It is relatively uncommon to actually trace pathways all the way back to identify how toxicant exposures, to individual toxicants or to mixtures, could lead to disturbances in these molecular regulatory systems.

At present, there has been no clear explanation for the differences reported in epidemiological studies for POP exposures and T2D. The apparent inconsistencies may be related to the idea that POPs are involved in the pathogenesis of T2D by interfering with endocrine signaling pathways. Low-dose effects have been proposed as possible biological responses to POPs as endocrine disruptors (Vandenberg et al. 2012). Endocrine function generally declines with age because hormone receptors become less sensitive, and levels of most hormones change with age (Chahal and Drake 2007). Therefore, the different age distributions among study populations might have led to the different results, even when similar concentrations of POPs were compared. In addition, the endocrine-disturbing effects of a specific POP might differ relative to the presence and concentrations of other potential endocrine disruptors. Inconsistences across studies may also have been caused by the underlying risk (nutrition, polymorphism, non-chemical stressors, and diseases) as well as by the endocrine state (sex, menopausal status) in the study population. Humans are exposed to a mixture of various POPs, and exposure patterns are unique to each study population. Although concentrations of a particular POP might be similar between two populations, the strength of association between that POP and diabetes can differ depending on the concentrations of other POPs. The POPs investigated in the present study are lipophilic and have similar pharmacokinetic behavior in the body, which means they have the potential to interact and to influence the overall joint toxicity, so they should be considered as mixtures instead of on an individual basis. Hence, we need highly sophisticated data analysis tools to correlate multi-chemical POP exposure and the health effects observed in epidemiological studies.

Novel methods of analysis including machine learning, bioinformatics, and systems biology tools are available and can be used to identify specific outcome pathways from complex data (Minihane et al. 2015). These technologies can help identify specific and sensitive biomarkers, as proposed in our study. This type of cluster identification of biomarkers as signatures of exposure to chemical mixtures could help advance the development of methods of mixtures risk assessment. Epidemiological studies need to assess inflammatory markers related to metabolic diseases; therefore, the sensitivity and specificity of these available biomarkers, which are influenced by a range of modifying factors (chemical mixture components, age, sex, diet, disease, gut biota, etc.), can be studied using multiple sophisticated techniques. Innovative markers of inflammation could be developed for use in human population studies and disease prevention and for clinical use to detect multiple chemical exposures.

The resulting global network of common downstream activation targets was significantly enriched with metabolic disease–related targets. Interestingly, neoplasms were also over-represented among the common targets, with transcription factors, receptor tyrosine kinases, and cyclin genes identified by our queries. This common pathway could guide our understanding of the potential carcinogenic mechanisms shared by the POPs.

The final network presents a novel systems biology and toxicology model of different molecular mechanisms of POP action that point to common disease outcomes. Future experimental evaluation of this model might lead to the development of new predictive markers of POP effects that could translate into new strategies for disease prevention and clinical use. Specific avenues of laboratory research might include, but are not limited to, *in vitro* studies of target cell populations such as liver cells and adipocytes; moreover, cell-line studies can be performed using pancreatic cells, hepatocytes, and brown adipocytes. Complementary *in vivo* studies in both normal and obese mouse strains dosed with POPs could be performed to determine whether the observed *in vitro* study findings are also observed after *in vivo* exposure. In addition, studies could be performed using transgenic mouse models with human fatty acid metabolism genes as well as with other potential monogenic or polygenic rodent models. Both *in vitro* and *in vivo* studies should be conducted using exposure to the three selected POPs on an individual or mixture basis using a factorial design approach. Specific receptors or pathway nodes of interest identified using these combined *in silico* laboratory model approaches could be technically evaluated using genomic, proteomic, or metabolomic methods. Putative biomarkers identified by these combined approaches could be further developed/translated into commercial test kits for clinical applications.

## Conclusion

We examined three representative POPs and their potential combined effects via possible protein–protein interactions. Our results, obtained using the inflammatory biomarkers pathway, showed that looking beyond the pathway for an individual chemical reveals a complex network of pathways that could be the basis of a mechanism of joint toxicity of mixtures. Hence, the body burden of chemical mixtures, particularly mixtures of lipophilic chemicals such as POPs, should be considered within the larger framework of diabetes, metabolic syndrome, and other chronic diseases. Biomarkers identified through such pathway analyses could be studied thoroughly and used to test real-world mixture exposures. Further investigations of the influence of factors such as multiple chemical exposures, nutrition, age, sex, and genetic variations will help develop personalized, specific treatment protocols for these complex diseases.
